# Placental Genome and Maternal-Placental Genetic Interactions: A Genome-Wide and Candidate Gene Association Study of Placental Abruption

**DOI:** 10.1371/journal.pone.0116346

**Published:** 2014-12-30

**Authors:** Marie Denis, Daniel A. Enquobahrie, Mahlet G. Tadesse, Bizu Gelaye, Sixto E. Sanchez, Manuel Salazar, Cande V. Ananth, Michelle A. Williams

**Affiliations:** 1 Department of Epidemiology, Harvard School of Public Health, Boston, Massachusetts, United States of America; 2 UMR AGAP (Amélioration Génétique et Adaptation des Plantes méditerranéennes et tropicales), CIRAD, Montpellier, France; 3 Center for Perinatal Studies, Swedish Medical Center, Seattle, Washington, United States of America; 4 Department of Epidemiology, School of Public Health, University of Washington, Seattle, Washington, United States of America; 5 Department of Mathematics and Statistics, Georgetown University, Washington, D.C., United States of America; 6 Sección de Post Grado, Facultad de Medicina Humana, Universidad San Martín de Porres, Lima, Peru; 7 A.C. PROESA, Lima, Peru; 8 Department of Obstetrics and Gynecology, San Marcos University, Lima, Peru; 9 Department of Obstetrics and Gynecology, College of Physicians and Surgeons, Columbia University Medical Center, New York, New York, United States of America; 10 Department of Epidemiology, Mailman School of Public Health, Columbia University, New York, New York, United States of America; Indiana University Bloomington, United States of America

## Abstract

While available evidence supports the role of genetics in the pathogenesis of placental abruption (PA), PA-related placental genome variations and maternal-placental genetic interactions have not been investigated. Maternal blood and placental samples collected from participants in the Peruvian Abruptio Placentae Epidemiology study were genotyped using Illumina’s Cardio-Metabochip platform. We examined 118,782 genome-wide SNPs and 333 SNPs in 32 candidate genes from mitochondrial biogenesis and oxidative phosphorylation pathways in placental DNA from 280 PA cases and 244 controls. We assessed maternal-placental interactions in the candidate gene SNPS and two imprinted regions (IGF2/H19 and C19MC). Univariate and penalized logistic regression models were fit to estimate odds ratios. We examined the combined effect of multiple SNPs on PA risk using weighted genetic risk scores (WGRS) with repeated ten-fold cross-validations. A multinomial model was used to investigate maternal-placental genetic interactions. In placental genome-wide and candidate gene analyses, no SNP was significant after false discovery rate correction. The top genome-wide association study (GWAS) hits were rs544201, rs1484464 (CTNNA2), rs4149570 (TNFRSF1A) and rs13055470 (ZNRF3) (p-values: 1.11e-05 to 3.54e-05). The top 200 SNPs of the GWAS overrepresented genes involved in cell cycle, growth and proliferation. The top candidate gene hits were rs16949118 (COX10) and rs7609948 (THRB) (p-values: 6.00e-03 and 8.19e-03). Participants in the highest quartile of WGRS based on cross-validations using SNPs selected from the GWAS and candidate gene analyses had a 8.40-fold (95% CI: 5.8–12.56) and a 4.46-fold (95% CI: 2.94–6.72) higher odds of PA compared to participants in the lowest quartile. We found maternal-placental genetic interactions on PA risk for two SNPs in PPARG (chr3∶12313450 and chr3∶12412978) and maternal imprinting effects for multiple SNPs in the C19MC and IGF2/H19 regions. Variations in the placental genome and interactions between maternal-placental genetic variations may contribute to PA risk. Larger studies may help advance our understanding of PA pathogenesis.

## Introduction

Placental abruption (PA), the premature separation of the placenta from the uterine wall prior to delivery of the fetus, complicates about 1% of pregnancies and is an important cause of maternal and neonatal morbidity and mortality [1]–[7]. Evidence from studies conducted during the last three decades suggests that hypertensive disorders, advanced maternal age, grand-multiparity, thrombophilia, cigarette smoking, illicit drug use and external trauma to the abdomen are associated with an increased risk of PA [8]–[13]. In addition, other putative risk factors have also been recently described for PA, including maternal iron deficiency anemia, hyperhomocystinemia, mood and anxiety disorders, migraine and headache disorders, maternal infection and/or inflammation [4], [14]–[21]. Pathophysiologic mechanisms involved in PA include uteroplacental underperfusion, chronic hypoxemia, uteroplacental ischemia and infarctions, and thrombosis [5], [13], [22]–[23].

As a multi-factorial disorder of complex origin, PA aggregates in families of women with the condition [24], suggesting a strong role for genetic predisposition, a thesis supported by a number of candidate gene studies [25]–[29]. Findings from recent PA-related genome-wide association studies (GWAS) and candidate gene association studies (mitochondrial biogenesis and oxidative phosphorylation pathway genes) in the maternal genome by our group provided suggestive evidence supporting associations of variation in maternal cardiometabolic genes with risk of PA [30], [31]. Given these findings indicative of the importance of genetic susceptibility factors in PA and the evidence highlighting the central role of placental pathology in PA [5], [28], [32], we hypothesized that genetic variations in the placental genome, particularly those variants in mitochondrial biogenesis (MB) and oxidative phosphorylation (OP) pathways, are associated with PA risk. Further, using data from both maternal and placental genomes, we examined interactions between placental and maternal genetic variations (in MB-OP pathway genes and imprinted regions) on risk of PA.

## Materials and Methods

### Study Setting and Study Population

The current study was conducted in the setting of the Peruvian Abruptio Placentae Epidemiology (PAPE) study that has been described before [30], [31]. Briefly, PAPE study participants were recruited and enrolled among patients admitted for obstetrical services to the Hospital Nacional Dos de Mayo, Instituto Especializado Materno Perinatal, and Hospital Madre-Niño San Bartolomé in Lima, Peru, between August 2002 and May 2004 and between September 2006 and September 2008. Hospital admission and delivery logs were monitored daily to identify PA cases among new admissions to antepartum, emergency room, and labor and delivery wards of participating hospitals. PA was diagnosed based on evidence of retroplacental bleeding (fresh blood) entrapped between the decidua and the placenta or blood clots behind the placenta and any two of the following: (i) vaginal bleeding in late pregnancy not due to placenta previa or cervical lesions; (ii) uterine tenderness and/or abdominal pain; and, (iii) non-reassuring fetal status or death. Controls were selected from among pregnant women who delivered at participating hospitals during the study period and did not have a diagnosis of PA in the current pregnancy. For the current study, investigating associations of placental genome with risk of PA, 280 PA cases and 244 controls who provided placental samples at delivery were included. A subset of these cases and controls that also provided blood samples (222 PA cases and 198 controls) were also included in the maternal-placental genetic interaction investigations.

Ethical approval for the study was granted by the Institutional Review Boards (IRB) of Hospital Nacional Dos de Mayo, Instituto Especializado Materno Perinatal, Hospital Madre-Niño San Bartolomé in Lima, Peru and the IRB of Swedish Medical Center, Seattle, WA. All participants provided written informed consent in accordance with the principles of the declaration of Helsinki.

### Data collection

Standardized structured questionnaires administered by trained research personnel were used to collect information on socio-demographic characteristics (including maternal age, marital status, employment status during pregnancy, and smoking and alcohol consumption before and during pregnancy), and medical history. A brief physical examination was conducted to measure maternal height, weight, and mid-arm circumference. Medical records were reviewed to abstract information on course and outcomes of the pregnancy. At delivery, placental samples were collected for DNA extraction and genotyping as described below.

### Placental sample collection, DNA extraction, and genome-wide genotyping

Placentas were collected immediately after delivery. Placentas were weighed, double bagged and transported in coolers. The chorionic plate and overlying membranes were stripped and tissue biopsies (approximately 0.5 cm^3^ each) were obtained from 8 sites (4 maternal and 4 fetal). For this study, biopsy samples taken from the fetal side were sampled for genomic DNA extraction. Biopsies were placed in cryotubes, snap frozen in liquid nitrogen, and stored at −80°C until analysis. The Gentra PureGene Cell kit for DNA preparations (Qiagen, Hilden, Germany) was used to extract DNA from placental samples. Genotyping was conducted using the Illumina Cardio-Metabochip (Illumina Inc, San Diego, CA) platform [30], a high-density custom array designed to include 217,697 SNPs that represent DNA variations at regions previously related to diseases and traits relevant to metabolic and atherosclerotic-cardiovascular endpoints [33], [34]. During the assay manufacturing process 20,972 SNPs (9.6%) failed, resulting in 196,725 SNPs available for genotyping, downstream quality control and statistical analyses [34], [35].

### Candidate gene/SNP selection

For the candidate association study, 32 genes that were involved in mitochondrial biogenesis and oxidative phosphorylation were selected based on literature [31] and a total of 333 SNPs belonging to these genes and found in the Cardio-MetaboChip were included in the candidate gene association analyses. For the maternal-placental genetic interaction study, 325 of these SNPs that also passed quality control in maternal blood genomes, as well as SNPs in imprinted regions (5 SNPs in IGF2/H19 and 33 SNPs in C19MC) included in the Cardio-MetaboChip were analyzed.

### Data quality control

Quality control and preprocessing were performed on the genotype data. Individuals with genotyping failure in more than 10% of SNPs were removed (n = 2). SNPs with minor allele frequency (MAF) less than 1% or that failed to be genotyped in more than 10% of the study samples were removed (n = 77, 276), as well as SNPs not in Hardy-Weinberg equilibrium (HWE) among controls (n = 667). After these quality control procedures, a total of 118,782 genome-wide SNPs were examined among 280 PA cases and 244 controls. Similar quality control procedures were performed on the maternal blood genotype data, resulting in 222 PA case and 198 controls among maternal-placental pairs.

### Statistical analyses

Univariate logistic regression model was used to estimate odds ratio (OR) and 95% confidence interval (95% CI) relating each SNP with risk of PA, in the genome-wide and candidate gene analyses. For multiple testing correction, a false discovery rate (FDR) procedure was used [36]. Functions and functional relationships of genes represented by the top 200 genome-wide SNPs were obtained by pathway analysis using the Ingenuity Pathway Analysis (IPA, Ingenuity Systems, www.ingenuity.com) software. Gene-enrichment network score based on a modified Fisher's exact test were calculated to rank biological significance of networks in relation to PA.

In multivariable analyses, we applied penalized logistic regression models to identify sets of SNPs that are jointly associated with the risk of PA. These penalized approaches have previously been applied in the context of GWAS and have shown promising results [37]–[38]. These methods allow the selection of relevant variables or groups of variables and the estimation of their regression coefficients [39]. The number of selected variables is guided by a penalty parameter: the larger the parameter, the smaller the selected subset. A 20-fold cross-validation approach was performed to select the penalty parameter and the value yielding the smallest prediction error was used. For the genome-wide SNP analysis, we applied a lasso regression [39]. One characteristic of lasso regression is that it selects a single variable among a set of correlated variables. To circumvent this, SNPs in high linkage disequilibrium with a selected SNP were also considered using an r^2^ threshold of 0.8 within 500 kb. For SNPs in the candidate gene analyses, a group penalty approach was used to account for the membership in a gene [40]. Furthermore, we considered a bi-level selection approach that uses a composite minimax concave penalty [41], [42] to select candidate genes associated with PA as well as relevant SNPs within those genes. These penalized regression methods do not accommodate missing values and the software BEAGLE version 3.3.2 [43] was used to impute missing genotypes.

For weighted genetic risk score (WGRS) analyses [44], a 10-fold cross-validation procedure was implemented to protect against model over-fitting, which arises from using the same data to estimate the regression parameters used in computing WGRS and to evaluate the association between PA risk and WGRS [45]. The procedure consisted of randomly partitioning the data into 10 equal size subsamples, using nine of the subsamples as training set and the left-out one as validation set, with each subsample being used in turn as a test set. For each fold, a multivariate logistic regression model was fit on the training set using the SNPs selected from multivariate analyses. A weighted approach was then used to compute Genetic Risk Scores (GRS) in the validation set by multiplying the number of risk allele for each locus by its associated effect size estimated from the training set. Once the WGRS were obtained for all individuals, the subjects were categorized into four groups defined by the quartiles in the control. A logistic regression model was then fit to examine the association of the WGRS with PA risk using the lowest quartile (Group 1) as a reference and adjusting for infant sex and population admixture. This 10-fold cross-validation procedure was repeated 1000 times to account for the variability in randomly partitioning the data into subsamples. The receiver operating characteristics (ROC) curve for each of the replicates was evaluated. The estimated effect sizes and AUCs over the 1000 replicates were used to obtain the respective point estimates and confidence intervals.

Maternal-placental interaction analyses (for candidate genes and imprinted regions) were performed using a multinomial model proposed by [46] and implemented in the EMIM and PREMIM software tools [47]. The method requires some biological assumptions, such as Hardy-Weinberg equilibrium (HWE), random mating, and rare disease. For each SNP, four models were considered and a model selection procedure based on the Bayesian information criterion (BIC) was applied. The four models correspond to allele effects operating only at the fetal level (Model F), allele effects operating only at the maternal level (Model M), an additive effect of maternal and fetal effects (Model M+F), and a model that includes a maternal-placental interaction effect (Model I). For the latter, we used a parametrization that introduces two interaction terms capturing incompatibility between maternal and placental genotypes; the interaction effects operate when the infant has one copy and the mother has either zero or two copies of the risk allele [46], [48]. Maternal imprinting effect, which corresponds to the factor multiplying the disease risk if the infant inherits a risk allele from the mother, was tested using a likelihood ratio test [46], [49].

Adjustment for the first four principal components was done for all univariate and multivariable logistic regression models to take into account population stratification. The various statistical analyses were conducted using a combination of software tools: PLINK, PREMIM, EMIM, Haploview, and R. As for multivariable approaches the R packages ncvreg and grpreg were used [41], [50]. The pathway analyses were conducted using the Ingenuity Pathway Analysis (IPA) software.

## Results

Table 1 shows selected characteristics of PA cases and controls. Average maternal age in both groups was around 27 years. Alcohol use during pregnancy and preeclampsia/eclampsia were more common among PA cases than controls. As expected, infant birthweight and gestational age at delivery were lower for PA cases compared with controls.

**Table 1:** Socio-demographic and reproductive characteristics and infant outcomes in the study sample, Lima, Peru.

Maternal Characteristics	Placental Abruption		
	Cases	Controls	
	(n = 280)	(n = 244)	p-value**
**Maternal age at delivery, years** *	27.03 (6.5)	27.3 (6.6)	0.517
** <35**	239 (85%)	200 (82%)	0.337
** ≥35**	40 (14%)	43 (18%)	
**Nulliparous**	115 (41%)	95 (39%)	0.655
**Less than High school education**	220 (79%)	186 (76%)	0.598
**Employed during pregnancy**	126 (45%)	108 (44%)	0.935
**Planned pregnancy**	114 (41%)	99 (.41%)	0.929
**No prenatal vitamin**	82 (29%)	65 (27%)	0.559
**Smoked during pregnancy**	12 (4%)	5 (2%)	0.216
**Alcohol use during pregnancy**	9 (3%)	0 (0)	0.004
**Pre-pregnancy BMI, kg/m2** *	23.5 (3.5)	23.9 (3.9)	0.228
** <18.5**	14 (5%)	8 (3%)	
** 18.5–24.9**	179 (64%)	149 (61%)	
** 25.0–29.9**	57 (2%)	56 (23%)	
** ≥30.0**	13 (5%)	18 (7%)	
**Chronic hypertension**	8 (3%)	4 (2%)	0.391
**Preeclampsia or Eclampsia**	58 (21%)	29 (12%)	0.005
**History of placental abruption**	2 (7%)	0 (0)	0.498
**Infant birthweight, grams** *	2357 (888)	3058 (825)	2.20E-16
**Gestational age at delivery, weeks** *	35 (4.3)	37.8 (3.5)	2.68E-15

*Mean (standard deviation), otherwise count (%).

**t-test and chi-square test respectively used for continuous and categorical variables.

**Figure 1:**
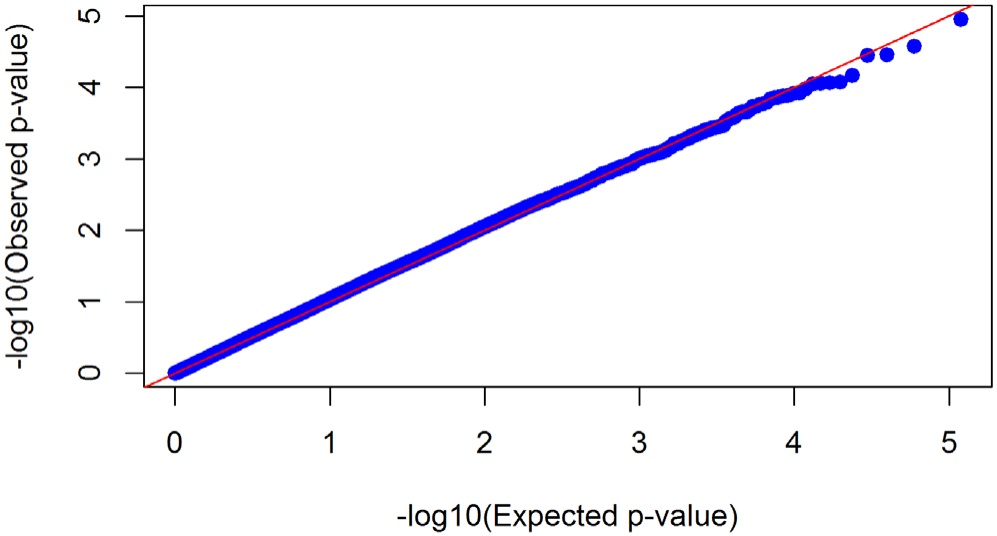
Quantile-Quantile plot. Quantile-quantile plot (QQ-plot) of raw p-values from univariate GWAS analysis adjusting for population stratification (genomic inflation factor λ = 1.168).

**Figure 2:**
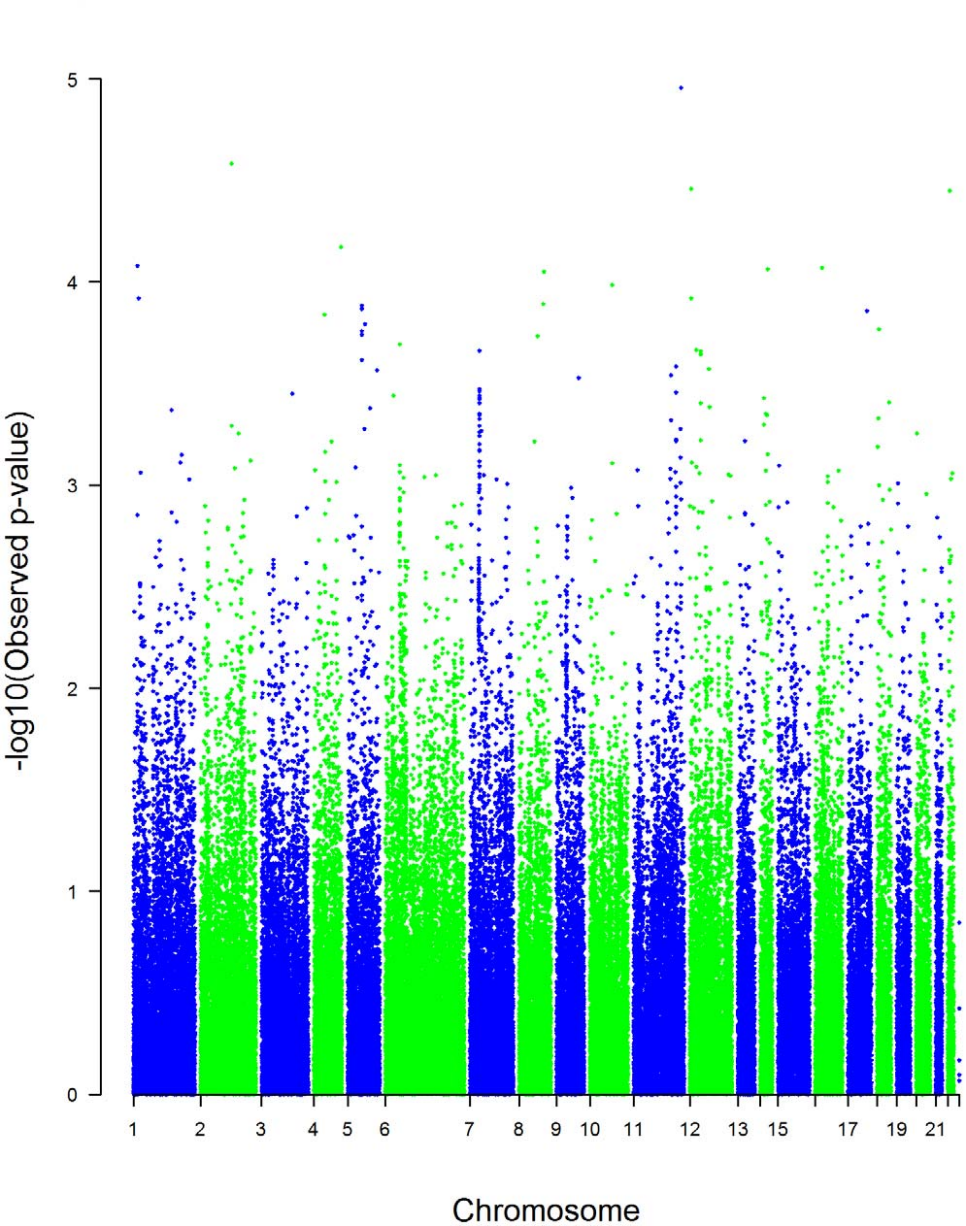
Manhattan plot. Manhattan plot of raw p-values from univariate GWAS analysis adjusting for population stratification.

**Figure 3:**
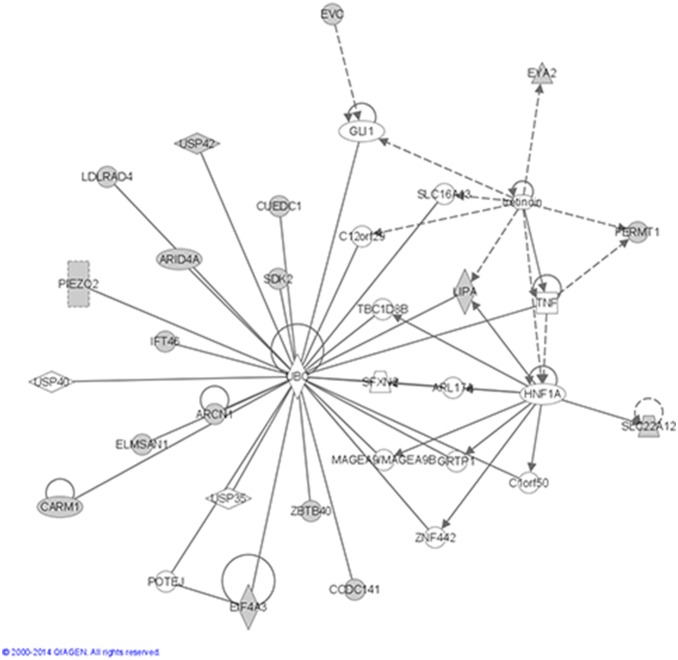
Molecules in the top network. Representation of molecules in the top network enriched by genes corresponding to the top 200 SNPs from univariate GWAS analyses.

In the GWAS analyses, we did not observe significant genomic inflation (λ = 1.17) (Fig. 1). None of the FDR-corrected p-values were lower than a 0.05 threshold (Table 2). The top GWAS hits were *rs544201*, *rs1484464* (CTNNA2), *rs4149570* (TNFRSF1A) and *rs13055470* (ZNRF3) (nominal p-values: 1.11e-05 to 3.54e-05) (Fig. 2 and Table 2). Functions of 56 genes represented by the top 200 SNPs from univariate GWAS analyses were examined using IPA. The top five networks from these analyses with p-values less than 0.05 are shown in Table 3. The top network enriched by these genes was a network of cell cycle, growth and proliferation (score = 43, p-value = 2.12e-19) (Fig. 3). In the candidate gene analyses, none of the FDR-corrected p-values were lower than a 0.05 threshold. The top hits in these analyses were *rs16949118* (COX10) and *rs7609948* (THRB) (nominal p-values: 6.00e-03 and 8.19e-03, respectively) (Table 4). In addition, several SNPs in the PPARG gene were among the top hits in the candidate gene analyses.

**Table 2:** Top 20 hits from univariate analyses examining genome-wide genetic variations related to placental abruption risk.

NCBIGene Name	SCANGene Name	SNP	MinorAllele	MAF	OR (95% CI)	Empiricalp-value
		rs544201	T	0.13	0.33 (0.21–0.54)	1.11E-05
CTNNA2	CTNNA2	rs1484464	G	0.3	1.80 (1.37–2.38)	2.62E-05
TNFRSF1A	TNFRSF1A	rs4149570	A	0.21	1.88 (1.40–2.53)	3.46E-05
ZNRF3	ZNRF3	rs13055470	A	0.31	0.52 (0.39–0.71)	3.54E-05
ACSL1	LOC11394	rs9997745	A	0.03	3.78 (1.97–7.26)	6.73E-05
		rs10754855	A	0.26	0.52 (0.38–0.72)	8.34E-05
		rs3096425	G	0.47	0.58 (0.44–0.76)	8.50E-05
		rs12896434	A	0.19	1.84 (1.36–2.49)	8.61E-05
		rs2436893	A	0.3	1.72 (1.31–2.25)	8.86E-05
LIPA	LIPA	rs7922269	A	0.44	0.58 (0.43–0.76)	1.03E-04
SCNN1A	SCNN1A	rs2228576	A	0.19	1.83 (1.34–2.48)	1.20E-04
ZBTB40	ZBTB4	rs12725956	G	0.08	2.24 (1.49–3.38)	1.20E-04
		rs3133572	A	0.15	0.44 (0.29–0.67)	1.28E-04
ZBED3-AS1		rs4457053	G	0.45	0.59 (0.46–0.78)	1.31E-04
ZBED3-AS1		rs7708285	G	0.45	0.59 (0.46–0.78)	1.36E-04
SDK2		rs9913193	G	0.25	0.54 (0.39–0.74)	1.38E-04
ADAMTS3	ADAMTS3	rs4694121	A	0.55	0.60 (0.46–0.78)	1.45E-04
		rs6871240	A	0.18	1.8 (1.33–2.46)	1.62E-04
PIEZO2		rs9964303	A	0.3	0.57 (0.42–0.76)	1.70E-04
		chr5∶76460816	C	0.45	0.60 (0.46–0.78)	0.000174

Abbreviations: MAF = Minor Allele Frequency in Controls.

**Table 3:** Significant networks represented by top GWAS hits.

Genes from GWAS analysis with adjustment for the first four components					
ID	Molecules inNetwork	Score	FocusMolecules	Top Diseasesand Functions	P-value
1	**ARCN1,ARID4A**,ARL17A,C12orf29,C1orf50,**CARM1**,**CCDC141,CUEDC1,EIF4A3,ELMSAN1,EVC,EYA2,FERMT1**,GLI1,GRTP1,HNF1A,**IFT46,LDLRAD4,LIPA**,MAGEA9/MAGEA9B,**PIEZO2**,POTEJ,**SDK2**,SFXN2,SLC16A13,**SLC22A12**,TBC1D8B,TNF,tretinoin,UBC,USP35,USP40,**USP42,ZBTB40**,ZNF442	43	18	Cell Cycle,Cellular Growthand Proliferation,Gene Expression	2.12E-19
2	**ACSL1**,Akt**,ANGPTL4**,ARAP3,**ARHGAP26**,Cyp2j9,Focal adhesion kinase,ganglioside GD2**,GLIS3**,**GYS2**,IL22R1-IL10R2,Immunoglobulin,INSRR,Insulin,**IPO8,LPP**,miR-491–5p (and other miRNAs w/seed GUGGGGA),**NCOA7**,NFkB (complex),NRG4,NTN4,P38 MAPK,PID1,PKN3,**PTPRB,RAB31,RGS5,SCNN1A**,SLC2A5,SLC30A7,SLC35B2,SLC3A1,**TNFRSF1A**,TRAF1-TRAF2-TRAF3,WISP3	28	13	Endocrine SystemDevelopment andFunction, TissueMorphology,Cellular Development	6.91E-13
3	ADAMDEC1,**ADAMTS3**,AKT2,**ART3**,**ATP10A**,beta-estradiol,Ca2+,Calmodulin-Camk4a2+,CCDC82,Cetn4,COA4,CPA2,**CTNNA2**,**DGKB,DOK6**,ERBB2,FURIN,GDPD3,HDAC4,HNF4A,Interferon alpha,**INTS4,KCNIP1**,MAPK1,MITF,NUDT6,**PDE11A**,PRR15L,**RHOBTB2**,SLC24A3,**SPAG16**,Timd2,TMEM258**,TRPM1,ZNRF3**	28	13	Energy Production,Molecular Transport,Nucleic AcidMetabolism	6.91E-13
4	SYT14,**SYT16**	3	1	Hereditary Disorder,NeurologicalDisease, Cancer	4.98E-02
5	KIAA1524,**SLC12A8**	3	1	Cancer, CellularMovement,GastrointestinalDisease	4.98E-02

The networks were generated using Ingenuity Pathways Analysis (Ingenuity Systems, www.ingenuity.com). Each gene identifier was mapped to its corresponding gene object in the Ingenuity Pathways Knowledge Base (IPKB) and overlaid onto a global molecular network developed from information contained in the IPKB. Scores, corresponding to degree of enrichment, are negative log of p-values from Fisher’s exact test. Genes in bold (focus molecules) are genes that correspond to top hit SNPs in our genome-wide association study of placental abruption.

**Table 4:** Top 20 SNPs in univariate analyses of candidate genes in relation to risk of placental abruption.

SCAN Gene Name	SNP	MinorAllele	MAF	OR (95% CI)	Empirical p-value
**COX10**	rs16949118	A	0.09	1.74 (1.17–2.59)	0.006003
**THRB**	rs7609948	A	0.19	1.48 (1.11–1.99)	0.008191
**COX5A**	chr15∶73015771	G	0.01	2.80 (1.09–7.21)	0.03232
**THRB**	rs17787283	A	0.12	0.64 (0.42–0.97)	0.03637
**PRKCA**	rs3848426	A	0.29	1.33 (1.02–1.74)	0.03651
**PPARG**	chr3∶12388339	C	0.23	1.37 (1.015–1.848)	0.03935
**NDUFS4**	rs1388111	A	0.51	0.78 (0.61–1.01)	0.05589
**CAMK2B**	chr7∶44224020	A	0.33	1.31 (0.99–1.72)	0.05679
**CAMK2D**	rs4834348	A	0.18	0.70 (0.49–1.01)	0.05689
**PPARG**	rs11709077	A	0.26	1.31 (0.98–1.75)	0.06663
**NDUFC2-KCTD14**	rs627297	C	0.18	0.74 (0.53–1.03)	0.07345
**CAMK2B**	chr7∶44224468	A	0.33	1.28 (0.97–1.69)	0.07898
**THRB**	rs12639293	A	0.29	1.28 (0.97–1.69)	0.07978
**PPARG**	chr3∶12326521	C	0.26	1.29 (0.96–1.72)	0.08784
**PPARG**	rs4135275	G	0.28	0.78 (0.58–1.05)	0.0958
**NDUFS4**	rs2168662	G	0.45	0.80 (0.62–1.04)	0.09672
**TUFM**	chr16∶28763228	A	0.24	0.76 (0.55–1.06)	0.1012
**PPARG**	chr3∶12344401	A	0.26	1.27 (0.95–1.69)	0.1048
**PPARG**	chr3∶12352344	A	0.3	0.79 (0.59–1.05)	0.1053
**PPARG**	chr3∶12340308	A	0.26	1.27 (0.95–1.69)	0.1059

Abbreviations: MAF = Minor Allele Frequency in Controls.

Among 118,782 SNPs included in the GWAS analyses, six SNPs were selected using lasso regression (Table 5). All six SNPs were among the top 200 hits, including the top three hits, identified using the univariate logistic regression analyses. When fitting a multiple logistic regression model with the selected SNPs, all 6 SNPs had empirical p-values lower than 0.05. Using a group penalty and bi-level selection approach, we identified 22 SNPs (in 14 genes) among the >300 SNPs included in the candidate gene analyses (Table 6). In multiple logistic regression analysis that included these SNPs, 11 SNPs had empirical p-values less than 0.05 (Table 6). WGRS were computed using SNPs selected from the respective GWAS and candidate gene multivariable analyses (Table 7). Both WGRSs were significantly associated with risk of PA (p-values<0.001). Participants in the highest quartiles for cross-validated GWAS-based WGRS and candidate gene-based WGRS had a 8.4 (95% CI: 5.8–12.56) and a 4.46 (95% CI: 2.94–6.72) fold higher odds of PA compared with participants in the respective referent quartiles (quartile 1) adjusting for infant sex and population admixture. The cross-validated AUCs for the ROC curves were estimated to be 0.71 (95% CI: 0.69–0.73) for the GWAS-based WGRS and 0.67 (95% CI: 0.65–0.7) for the candidate gene-based WGRS, confirming that the WGRS models have good predictive ability (S1 Fig. and S2 Fig.).

**Table 5:** Multiple logistic regression using SNPs selected in lasso regression.

Gene	SNP	SNPs in high LD*	Minor Allele	MAF	Odds Ratio	Empirical P-value	rank in univariate GWAS
	rs544201		T	0,13	0.33 (0.26–0.56)	4,19E-05	1
**CTNNA2**	rs1484464		G	0,30	1.69 (1.46–2.26)	4,24E-04	2
**TNFRSF1A**	rs4149570	rs2228576	A	0,21	1.77 (1.53–2.41)	2,58E-04	3
	rs10754855		A	0,26	0.58 (0.49–0.81)	1,35E-03	6
**ARHGAP26**	rs17287593		G	0,01	4.55 (2.99–10.67)	5,04E-04	47
**IFT46**	rs2277292	rs2277295; rs17122278	A	0,05	0.23 (0.14–0.6)	2,55E-03	60

*R^2^>0.8 within 500 kb.

**Table 6:** Multiple logistic regression based on SNPs selected from candidate genes using a bi-level selection approach.

Gene	SNP	Minor Allele	MAF	Odds Ratio	Empirical P-value
PPARG	chr3∶12388339	C	0.23	1.68 (1.21–2.34)	2.19E-03
THRB	rs7609948	A	0.19	1.6 (1.17–2.18)	3.31E-03
NDUFA10	rs6437237	A	0.48	0.63 (0.45–0.87)	5.73E-03
NDUFA10	rs4149549	A	0.19	1.7 (1.15–2.51)	8.12E-03
COX10	rs16949118	A	0.09	1.77 (1.15–2.7)	8.72E-03
THRB	rs17787283	A	0.12	0.56 (0.35–0.88)	1.15E-02
NDUFS4	rs1388111	A	0.51	0.71 (0.55–0.93)	1.31E-02
NDUFA12	rs11107847	A	0.45	0.71 (0.54–0.94)	1.57E-02
PPARG	chr3∶12363563	C	0.14	1.59 (1.05–2.41)	2.75E-02
NR1H3	chr11∶47226512	A	0.3	1.39 (1.03–1.85)	2.86E-02
COX5A	chr15∶73015771	G	0.01	2.55 (1.05–6.22)	3.87E-02
CAMK2D	rs4834348	A	0.18	0.7 (0.47–1.02)	6.48E-02
NDUFC2-KCTD14	rs627297	C	0.18	0.73 (0.52–1.03)	7.41E-02
PPA2	rs2298733	C	0.17	0.72 (0.5–1.03)	7.58E-02
PRKCA	rs3848426	A	0.29	1.27 (0.96–1.69)	9.95E-02
PPARG	chr3∶12412978	A	0.03	1.85 (0.87–3.93)	1.07E-01
COX5A	chr15∶73011246	A	0.17	0.74 (0.51–1.09)	1.31E-01
PRKCA	chr17∶61760907	G	0.34	1.23 (0.92–1.64)	1.56E-01
THRB	rs17014418	G	0.02	0.5 (0.17–1.42)	1.94E-01
COX7B2	rs17598636	G	0.02	0.54 (0.19–1.53)	2.45E-01
NR1H3	chr11∶47233666	A	0.07	0.76 (0.44–1.32)	3.33E-01
CAMK2B	chr7∶44227306	A	0.02	1.36 (0.5–3.68)	5.47E-01

**Table 7:** Association between risk of placental abruption and weighted genetic risk score (WGRS) computed from SNPs selected in multivariable analyses using repeated 10-fold cross-validations.

Weighted Genetic Risk Score (GRS)					
	1st Quartile	2^nd^ Quartile	3^rd^ Quartile	4^th^ Quartile	p-value
	Genome-wide Association Analysis*				
**OR** **	1	**2.53**	**3.78**	**8.40**	**<0.001**
**(95% CI)**	(Ref.)	**(1.47–4.71)**	**(2.2–6.4)**	**(5.8–12.56)**	

	Candidate Gene Analysis*				
**OR** **	1	**1.91**	**2.79**	**4.46**	**<0.001**
**(95% CI)**	(Ref.)	**(1.1–3.34)**	**(1.66–4.65)**	**(2.94–6.72)**	

*Cross-validated WGRS computed from SNPs selected from multivariable analyses.

**Cross-validated odds ratios (and 95% confidence intervals) from logistic regression models adjusted for sex, and population admixture, p-values associated to chi-square global test.

We observed evidence for maternal-placental genetic interaction (on PA risk) for 23 candidate SNPs (Table 8). In particular, maternal-placental genetic interaction on PA risk was found for two SNPs in PPARG (chr3∶12313450 and chr3∶12412978). The model selection procedure based on the BIC for the SNP chr3∶12412978, is shown in Table 9. The smallest BICcorresponds to Model I meaning that Model I fits the data best. In the imprinting effect analyses, we found that six SNPs in the C19MC region (of the 33 that were examined) and two SNPs in the IGF2-H19 region (of the five that were examined) showed evidence for maternal imprinting effect (empirical p-value <0.05) (Table 10 and Table 11). In addition, borderline imprinting effects were detected for a number of other SNPs in these regions.

**Table 8:** SNPs selected with maternal-placental interaction as best fitting model.

Gene	SNP
**PPARG**	chr3∶12412978
**PPARG**	chr3∶12313450
**PRKCA**	chr17∶61743445
**CAMK2B**	chr7∶44226231
**THRB**	rs12639293
**LRPPRC**	rs11899538
**COX5A**	chr15∶73008298
**COX5A**	chr15∶73012861
**COX5A**	chr15∶73001842
**COX5A**	chr15∶73008918
**PRKCA**	rs9896575
**THRB**	rs9809150
**PRKCA**	chr17∶61732949
**NR1H3**	chr11∶47233666
**PRKCA**	chr17∶61736374
**CAMK2B**	rs2075076
**CAMK2B**	rs1127065
**THRB**	rs2362186
**PRKCA**	chr17∶61735430
**PRKCA**	chr17∶61735623
**COX10**	rs16949118
**TRNT1**	rs7629889
**PPARGC1A**	rs12650562

**Table 9:** Sample model selection procedure for SNP chr312412978.

chr3∶12412978 Models compared	Loglikelihood of first model	Likelihood ratio test	BIC of the first model
**Model I vs Model M+F**	**−349.57**	**13.9504**	**726.0741**
**Model M+F vs Model F**	**−**356.546	22.9475	726.5577
**Model M+F vs Model M**	**−**356.546	19.13	726.5577
**Model M+F vs Null Model**	**−**356.546	34.423	726.5577
**Model M vs Model NULL**	**−**366.111	15.293	738.9543
**Model F vs Model NULL**	**−**368.019	11.4755	742.7718
**Model Null**	**−**373.757		

**Table 10:** Results of imprinting analysis for SNP mapping to C19MC.

C19MC site			
SNP	Position	Empirical p-value	Gene
rs12608629	57617596	0.101	
rs12985487	57757473	0.973	
rs12327640	57758705	0.089	
rs179320	57780245	0.293	ZNF701
**rs12974834**	**57855966**	**0.027**	**ZNF83**
rs12976870	57859746	0.1	ZNF83
rs4802981	57887238	0.564	ZNF83
rs4802987	57925085	0.059	ZNF611
rs4801931	57928058	0.351	ZNF611
rs10407762	57979913	0.543	ZNF600
rs12461390	57997054	0.809	ZNF600
**rs12982980**	**58041662**	**0.032**	**ZNF468**
**rs8112177**	**58108780**	**0.004**	**ZNF888**
rs7251313	58116136	0.118	ZNF888
rs12972202	58161816	0.926	
rs12610001	58162603	0.917	
rs1650966	58166272	0.899	ZNF702P
rs7258746	58246131	0.3	ERVV-2
rs10405102	58262679	0.715	ZNF160
rs7254015	58272963	0.655	ZNF160
rs17300167	58296859	0.537	ZNF160
**rs10423215**	**58340885**	**0.02**	**ZNF347**
rs4803058	58386953	0.786	ZNF665
rs11669754	58389180	0.45	ZNF665
rs6509732	58394320	0.628	
rs11084227	58419094	0.593	
**rs8100275**	**58442327**	**0.004**	**ZNF677**
rs2965261	58467498	0.676	
rs4263048	58469142	0.068	
**rs7250240**	**58614327**	**0.002**	**ZNF765**
rs7258566	58704092	0.128	
rs12982082	58717730	0.898	ZNF331
rs4994351	58724856	0.468	ZNF331

Rows in bold correspond to p-value<0.05.

**Table 11:** Results of imprinting analysis for SNP mapping to IGF2-H19.

IGF2-H19 site			
SNP	position	Empirical p-value	Gene
rs965912	1900778	0.061	TNNT3
rs6578974	2052309	0.199	
**rs7924768**	**2064648**	**0.038**	
**rs7926624**	**2085606**	**0.03**	
rs1004446	2126719	0.05	IGF2

Rows in bold correspond to p-value<0.05.

## Discussion

In this placental GWAS and candidate gene study of PA, no SNP had a significant association with PA risk following correction for false discovery. The top GWAS hits were *rs544201*, *rs1484464* (CTNNA2), *rs4149570* (TNFRSF1A), and *rs13055470* (ZNRF3). The top 200 SNPs of the GWAS were in genes of pathways involved in cell cycle, growth and proliferation. The top candidate gene hits were rs16949118 (COX10) and rs7609948 (THRB). Using repeated ten-fold cross-validations, participants in the highest quartiles of WGRS based on SNPs selected from GWAS and candidate gene analyses had 8.40-fold (95% CI: 5.8–12.56) and 4.46-fold (95% CI: 2.94–6.72) higher odds of PA compared to participants in the respective lowest quartiles. We also found evidence of maternal-placental genetic interaction on PA risk for two SNPs in PPARG (chr3∶12313450 and chr3∶12412978) and maternal imprinting effects for multiple SNPs in the C19MC and IGF2/H19 regions.

A number of studies have investigated genetic risk factors of PA. Most of these prior studies were candidate gene studies including investigations of thrombophilia and rennin-angiotensin system pathways, folate metabolism pathways, and interleukin receptor related pathways [24], [28], [29], [51]. Besides inconsistencies between reports of previous associations, few studies evaluated genome-wide variations and PA risk [24], [28], [29], [30], [31], [51]. Given the multi-factorial nature of PA pathogenesis, GWAS studies can potentially provide important information concerning possible genetic susceptibility factors and related novel pathways that play a role in occurrence of PA. To our knowledge, no prior study investigated PA risk and genetic variation in the placenta, where abnormal vasculature, thrombosis, lesions, and reduced perfusion, all culminate in the eventual occurrence of PA [5], [52].

In the current study, we did not have top hits of the GWAS that passed statistical significance after correcting for multiple testing. However, a number of SNPs among the top hits deserve mention. Of note, the top 10 SNPs represented five known genes including CTNNA2, TNFRSF1A, ZNRF3, ACSL1, and LIPA. The CTNNA2 gene codes for a protein linking cadherin adhesion receptors with the cytoskeleton. The SNP we identified in this gene, *rs1484464*, has been investigated in relation to a number of phenotypes, including tobacco use disorder, smoking cessation, coronary artery disease, lipid metabolism (HDL, total cholesterol, triglycerides) disorders, waist-hip ratio, CRP, glucose levels, insulin resistance, body mass index, blood pressure, and interleukin levels; however, significant associations have not been reported [53]–[62]. The TNFRSF1A is a gene member of the TNFR superfamily that activates the transcription factor NF-KB, mediates apoptosis, and functions as regulators of inflammation. The SNP we identified in the current study has been associated with susceptibility to inflammatory bowel disease, as well as response to anti-TNF treatment, in a large Danish cohort [63], [64]. It has also been associated with reduced expression of TNF alpha receptor [65].

While the ZNRF3 gene is encoding for an E3 ubiquitin-protein ligase that acts as a negative regulator of the Wnt signaling pathway and a tumor suppressor, no previous report, to our knowledge, exists on the ZNRF3 SNP we identified in the current study. The ACSL1 gene codes for an isozyme of the long chain fatty acid coenzyme A ligase family that converts free long-chain fatty acids into fatty acyl-CoA esters, a nuclear-encoded mitochondrial protein, and plays key role in lipid biosynthesis and fatty acid degradation. The *rs9997745* SNP in ACSL1 we identified has been previously associated with metabolic syndrome, fasting glucose, insulin levels, and insulin resistance [66]. Similarly, the LIPA gene encodes for lipase A, also known as cholesterol ester hydrolase, an enzyme that functions in the lysosome to catalyze the hydrolysis of cholesteryl esters and triglycerides. The SNP in LIPA, *rs792269*, is associated with total cholesterol, LDL cholesterol, and triglycerides [67]. None of the other top ten hits in our list had any previous associations reported. Our pathway analyses revealed that genes participating in cell cycle, cell growth and proliferation, and gene expression were overly represented by SNPs that were among the top hit of our GWAS. It is well known that disruptions in underlying normal placental growth and development are key underlying pathways that may later lead to the occurrence of PA [68].

The top hits in the candidate gene association analyses included SNPs in the COX10 (*rs16949118*) and THRB gene (*rs7609948*). COX10 encodes for the cytochrome C oxidase protein, the terminal component of the mitochondrial respiratory chain that catalyzes the electron transfer from reduced cytochrome C to oxygen. Genetic variations in this gene have been related to several diseases with underlying mitochondrial dysfunction including Alzheimers’ disease, neurodegenerative diseases and other childhood disorders [69]–[71]. The THRB gene encodes a protein that is a receptor for triidothyronine. Several studies have reported associations between disorders in thyroid (particularly low thyroid levels) and placental disorders including PA [72]–[74]. The SNPs identified in our study, however, have not been associated with phenotypes or clinical outcomes. In addition to these SNPs, several other SNPs in the PPARG gene, belonging to the PPAR-family of genes that have been well-described in relation to placental growth and development, were among the top hits in the candidate gene analyses [75], [76].

We examined associations between GRS, calculated from top hits of the GWAS and candidate gene analyses, respectively, and risk of PA and demonstrated strong associations between GRS and PA risk in both instances. While we did not use separate training and testing samples, we have used a cross-validation approach to protect against overfitting. These preliminary analyses are helpful to summarize identified effects of genetic variations, and could help in the construction of predictive models in future studies [77], [78]. In particular, such genetic prediction have advantages, over non-genetic prediction models, as they are highly stable over time and are more suited for assessment of lifetime risk. In fact, their utility improves over time [78]. In addition, the decreasing genotyping cost and minimal invasiveness associated with obtaining samples highlight the potential importance of genetic prediction scores. On the other hand, the need for large study populations, that comprise of training and testing sets, and identification of genetic variants that individually account for large effects, are potential challenges in this area of research.

In the current study, for two SNPs in PPARG (chr3∶12313450 and chr3∶12412978), models with maternal-genetic interaction on PA risk were found to fit the data best. A number of studies have previously reported interactions between maternal and fetal metabolic genes on maternal and fetal outcomes [79]–[84]. Liang et al. have previously reported significant interaction between maternal and fetal genetic variations at the G308A SNP of TNF-alpha on risk of preterm delivery [85]. Similarly, other investigators have also reported significant maternal-fetal genotype interactions in IL-1beta -511C/T genotype, 4845GG genotype of IL-1alpha, along with the G308A SNP of TNF-alpha on preterm delivery risk [84]. Sinsheimer et al. have particularly stressed that the complex interplay of maternal and fetal genetics can be important for phenotypes originating with the placenta, given the importance of both maternal and fetal related (paternal) risk factors to placenta-based diseases and demonstrated gene expression differences in placenta-related pathologies (e.g. preeclampsia) [83]. Interestingly, the gene that has been highlighted in relation to maternal genetic variation and fetal sex interactions on risk of gestational diabetes is the PPARG gene [81] More specifically, Hocher et al. have demonstrated that mothers carrying G alleles of the Pro12Ala polymorphism delivering a girl had a higher total glycated hemoglobin (6.81) versus mother carrying the same alleles but delivering boys (5.85) (p-value = 0.0015) [81]. However, to our knowledge, no prior study investigated maternal-placental genotype interactions in relation to PA risk. Similarly, placental growth and development is primarily under the control of fetal genes inherited from the father [5], [86]. Imprinted paternal alleles regulate formation of placenta and membranes surrounding the embryo, whereas the development of the embryo itself requires contribution from the maternally derived alleles [5], [86]. Our findings suggest maternal imprinting effects for multiple SNPs in the C19MC and IGF2/H19 regions. While these imprinting sites are well described for several conditions including placental growth and development, our findings are novel in terms of linking imprinting at these sites to PA risk [87], [88].

Given our sample size and related concerns regarding limited available statistical power, in exploratory analyses, we conducted maternal-placental interaction analyses of the PPARG gene using a haplotype-based approach. Tag SNPs from this gene were identified and haplotype blocks were defined using the Haploview software version 4.2 [89], [90]. A total of four haplotype blocks tagged by 29 SNPs were identified. For each haplotype block, three possible diplotypes HH, HH_0_, H_0_H_0_ as described in [80] were defined with the haplotype “H” denoting the “relevant” haplotype and “H_0_” denoting all other haplotypes. Each haplotype with frequency greater than 0.05 was considered as a potential “relevant” haplotype. For each “relevant” haplotype a procedure similar to the SNP interaction analysis was performed through the EMIM and PREMIM software tools. The “relevant” haplotype with the smallest BIC after evaluating all potential “relevant” haplotypes in each haplotype block was selected. This haplotype was called the optimal “relevant” haplotype. Findings from the exploratory interaction analyses are presented in Table 12. For haplotype block 1 a maternal-only effect was selected whereas for haplotype block 2 there was no evidence of haplotype effect. For haplotype block 3 a placental-only effect was selected. For both haplotype blocks (1 and 3), there was evidence that group 3 had a significantly lower risk of PA compared to the reference group (RR = 0.21, 95% CI: 0.066–0.68 and RR = 0.51, 95% CI: 0.293–0.878, respectively). For haplotype block 4, there was evidence of maternal-placental interaction effect. The group 3 showed a significantly higher risk of PA (RR = 5.71 95% CI: 3.398–9.604) whereas group 5 presented a significantly lower risk (RR = 0.18, 95% CI: 0.037–0.884) compared to the reference group. While findings from these haplotype based interaction analyses are encouraging, caution is warranted in interpreting these results due to uncertainties in the haplotype estimation process and the simplified model that considers diplotypes rather than evaluating all haplotypes using a reference.

**Table 12:** Results of haplotype-haplotype interaction analysis for haplotype blocks in PPARG gene.

	Haplotype block 1; optimal “relevant” haplotype denoted H	
Maternal Model	Relative Risk	CI (95%)
**gpRef {H_0_H_0__H_0_H_0_; H_0_H_0__H_0 _H}**	1	
**gp2 {H_0 _H_H_0_H_0_; H_0 _H_H_0 _H; H_0 _H_HH}**	0.6912	(0.386–1.237)
**gp3 {HH_H_0 _H; HH_HH}**	0.2123	(0.066–0.68)

	Haplotype block 3; optimal “relevant” haplotype denoted H	
Fetal Model	Relative Risk	CI (95%)
**gpRef {H_0_H_0__H_0_H_0_; H_0 _H_H_0_H_0_}**	1	
**gp2 {H_0_H_0__H_0 _H; H_0 _H_H_0 _H; HH_H_0 _H}**	1.0684	(0.812–1.406)
**gp3 {H_0 _H_HH_;_ HH_HH}**	0.5073	(0.293–0.878)

	Haplotype block 4; optimal “relevant” haplotype denoted H	
Interaction model	Relative Risk	CI (95%)
**gpRef {H_0_H_0__ H_0_H_0_}**	1	
**gp2 {H_0_H_0__H_0 _H}**	1.7656	(0.503–6.194)
**gp3 {H_0 _H_H_0_H_0_}**	5.7127	(3.398–9.604)
**gp4 {H_0 _H_H_0 _H}**	1.0177	(0.409–2.531)
**gp5 {H_0 _H_HH}**	0.1813	(0.037–0.884)
**gp6 {HH_H_0 _H}**	2.9148	(0.248–34.248)
**gp7 {HH_HH}**	1.0358	(0.168–6.404)

Diplotypes shown as maternal_placental.

This study expands the literature concerning the genetics of PA in a number of respects. We evaluated placental genetic variations, assessed interactions between maternal and placental genetic variations and examined placental imprinting effects on PA risk. However, several study limitations should be considered when interpreting our findings. First, our GWAS study has limited power to examine associations between genetic variations and disease risk, particularly for SNPs that have low to very low minor allele frequencies. Similarly, our statistical power to detect SNP-SNP interactions on PA risk was limited. To the extent possible we have tried to employ approaches that involve genetic risk scores to assess placental genetic variations contributions to PA risk. Second, many of the identified top hits of our analyses, both in the main effect and interaction models, have not been previously described either in genetic epidemiology or basic science investigations. Therefore, an important next step, along with replication efforts, should involve characterization of functional effects of these variations. Third, our imprinting effect assessment was based on mother-offspring dyad data and could benefit from triad data that also has information on fathers. Fourth, our GRS-based analyses, because of limited statistical power, did not involve independent training and testing sets, which would have been ideal for evaluating their predictive power, but rather relied on repeated ten-fold cross-validations. The WGRS will provide a summary estimate of effects of multiple SNPs, and can provide specific hypotheses that can be tested in future studies. Finally, findings from our study need to be cautiously generalized to other populations with different genetic make-up, population admixture, and environmental risk factors.

In sum, we found that variations in the placental genome and interactions between maternal-placental genetic variations may contribute to PA risk. We reported several novel loci where placental genetic variations may be associated with PA risk as well as several novel loci for maternal-placental genetic interactions on PA risk. Future larger investigations may help advance our understanding of PA pathogenesis.

## Supporting Information

S1 Fig **ROC curves for 1000 cross-validation replicates of the GWAS-based WGRS model with associated average AUC and 95% CI.**
(TIF)

S2 Fig **ROC curves for 1000 cross-validation replicates of the candidate gene-based WGRS with associated average AUC and 95% CI.**
(TIF)
