# Supplementary figures and images for: Placental Genome and Maternal-Placental Genetic Interactions: A Genome-Wide and Candidate Gene Association Study of Placental Abruption
Source: PLoS One. 2014 Dec 30;9(12):e116346. doi: 10.1371/journal.pone.0116346 (PMC4280220; doi:10.1371/journal.pone.0116346)

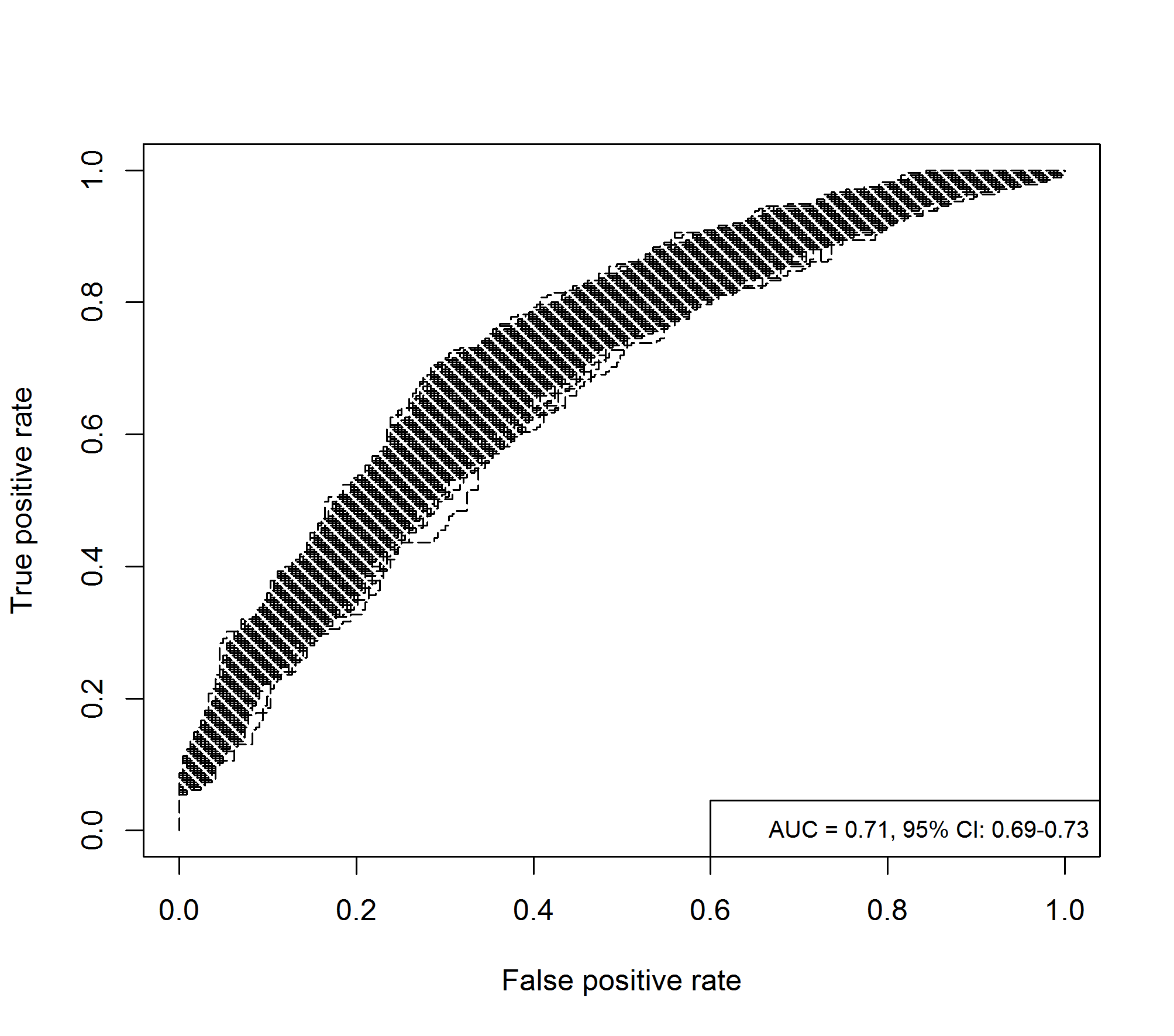

Supplement: S1 Fig — ROC curves for 1000 cross-validation replicates of the GWAS-based WGRS model with associated average AUC and 95% CI. (TIF) [file pone.0116346.s001.tif]

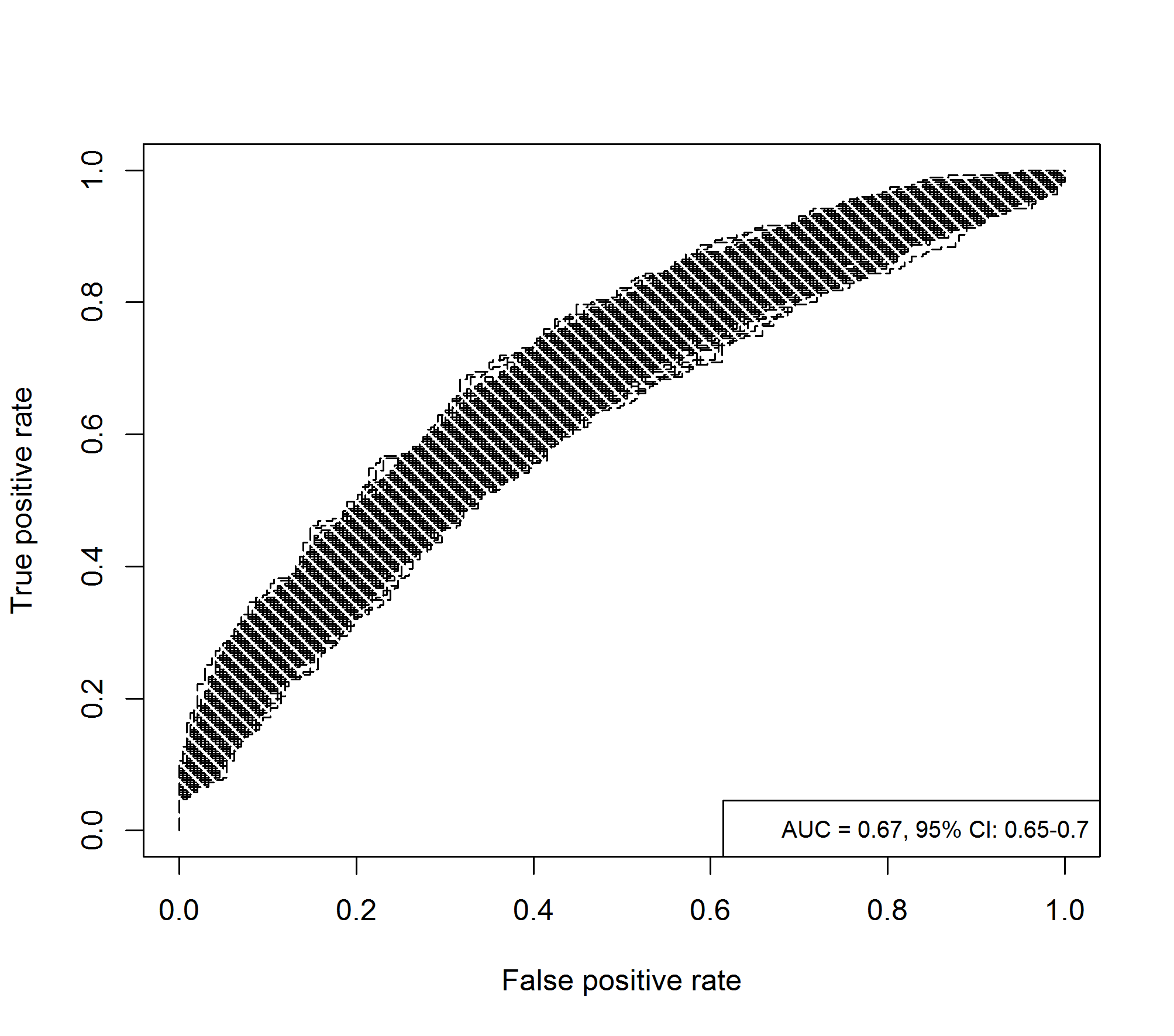

Supplement: S2 Fig — ROC curves for 1000 cross-validation replicates of the candidate gene-based WGRS with associated average AUC and 95% CI. (TIF) [file pone.0116346.s002.tif]
